# Dispersal by rodent caching increases seed survival in multiple ways in canopy‐fire ecosystems

**DOI:** 10.1002/ece3.2156

**Published:** 2016-05-30

**Authors:** N. B. Peterson, V. T. Parker

**Affiliations:** ^1^ Department of BiologySan Francisco State UniversitySan FranciscoCalifornia94132

**Keywords:** Arctostaphylos, chaparral, dispersal, scatter hoarding, seed bank

## Abstract

Seed‐caching rodents have long been seen as important actors in dispersal ecology. Here, we focus on the interactions with plants in a fire‐disturbance community, specifically *Arctostaphylos* species (Ericaceae) in California chaparral. Although mutualistic relationships between caching rodents and plants are well studied, little is known how this type of relationship functions in a disturbance‐driven system, and more specifically to systems shaped by fire disturbance. By burying seeds in the soil, rodents inadvertently improve the probability of seed surviving high temperatures produced by fire. We test two aspects of vertical dispersal, depth of seed and multiple seeds in caches as two important dimensions of rodent‐caching behavior. We used a laboratory experimental approach to test seed survival under different heating conditions and seed bank structures. Creating a synthetic soil seed bank and synthetic fire/heating in the laboratory allowed us to have control over surface heating, depth of seed in the soil, and seed cache size. We compared the viability of *Arctostaphylos viscida* seeds from different treatment groups determined by these factors and found that, as expected, seeds slightly deeper in the soil had substantial increased chances of survival during a heating event. A key result was that some seeds within a cache in shallow soil could survive fire even at a depth with a killing heat pulse compared to isolated seeds; temperature measurements indicated lower temperatures immediately below caches compared to the same depth in adjacent soil. These results suggest seed caching by rodents increases seed survival during fire events in two ways, that caches disrupt heat flow or that caches are buried below the heat pulse kill zone. The context of natural disturbance drives the significance of this mutualism and further expands theory regarding mutualisms into the domain of disturbance‐driven systems.

## Introduction

Scatter‐hoarding rodents have long been seen as important actors in dispersal ecology (e.g., Vander Wall 2010), both consuming and burying seed. The terms conditional mutualism and diffuse mutualism are often applied to this type of relationship, where net interaction results blur between antagonistic or mutualistic (Bronstein 1994; Stanton 2003; Thomson 2003; Forget et al. 2004). Seed dispersal effectiveness (Schupp et al. 2010) overall has largely been measured by distance from parent plants and the proportion of seed that survive to germination. But scatter hoarding has principally been interpreted for differential dispersal to safe sites that improve establishment for plants (e.g., Hanzawa et al. 1988; Hirsch et al. 2012). Critically, most research has focused on species with transient seed banks and ecosystems defined by infrequent, small disturbances, and long‐lived species (Stapanian and Smith 1978; Sork 1983; Smallwood et al. 2001; Sivy et al. 2011). Less understood is how the relationship functions with persistent seed bank species in systems shaped by chronic and large‐scale disturbance.

Scatter hoarding may be a critical process for seed survival in fire‐dominated shrublands (Moore and Vander Wall 2015; Parker 2015a). By caching seed, rodents disperse seeds vertically into the soil (Parker 2015a). Wildfires in these habitats burn above‐ground biomass and drive a heat pulse through the soil near the soil surface that may exceed the tolerance of buried seed, creating a kill zone near the surface. The soil kill zone is thought to be down to 2–5 cm in depth depending on fire intensity (Odion and Davis 2000; Keeley et al. 2014). Directed dispersal to safe sites in canopy‐fire vegetation could be described as spatially vertical placement into the soil below killing temperatures generated by fire rather than by the more typical horizontal movement (Moore and Vander Wall 2015; Parker 2015a).

In this study, we focus on a collection of plant species in the genus *Arctostaphylos* (Ericaceae) that develop persistent soil seed banks in vegetation characterized by a large‐scale, infrequent canopy‐fire regime. By caching seeds, scatter‐hoarding rodents inadvertently contribute to the persistent soil seed bank of these plants, potentially an important mechanism for the creation and structuring of seed banks. *Arctostaphylos* ecology also is intimately tied to fire regimes on the landscape. About two‐thirds of *Arctostaphylos* taxa die during a fire event (termed obligate seeders) where the remaining one‐third has the ability to re‐sprout after a fire (termed sprouters or facultative seeders) (Wells 1969). Both types rely on persistent soil seed banks with complicated germination triggers for long‐term persistence (Keeley 1977; Parker and Kelly 1989, 1990). Although *Arctostaphylos* germination is triggered by fire, mainly from chemicals derived from charred wood and delivered by precipitation, seed vulnerability to heat may begin at relatively low temperatures below 100°C (Keeley 1977; Odion and Davis 2000). This is quite low when compared to the range of temperatures typically experienced on the surface during a chaparral fire, from 200°C to 1000°C. Seeds survive these high surface temperatures, however, due to the insulating properties of soil. One study illustrates the extent to which soil reduces heat flow by determining that surface temperatures of 700°C were reduced to around 75°C at 5‐cm depth (DeBano 1988). Because most seed exhibit a shallow distribution in the soil, this steep heat gradient means minor differences of depth has great influence on seed survival.

The mechanisms that structure seeds banks and move seed down through the soil are not well understood and represent an important gap in our knowledge of *Arctostaphylos* ecology. Scatter‐hoarding rodents exhibit caching behavior with *Arctostaphylos* seed, suggesting that they play a role in structuring the seed bank (Parker 2015a). Furthermore, the high proportion of seedlings derived from caches after a high‐intensity fire suggests caching provides protection to seeds from high temperatures during a fire (Parker 2015a). Where most research investigating the mutualism between plants and scatter‐hoarding rodents typically is concerned with horizontal dispersal across the landscape; in a fire‐disturbance system, plant fitness depends on the vertical dispersal of seed into the soil and the structuring of soil seed banks.

The relationship between caching rodents and *Arctostaphylos* is complex, but introducing mutualism concepts to the relationship provides an effective framework. Expanding mutualism beyond tightly coevolved relationships and abandoning simple predator–prey models suggests conditions that determine the strength and direction of the relationship (e.g., Bronstein 2015). For example, we predict that cached seeds have a greater probability of surviving high temperatures than uncached seeds for two reasons. First, on average they are at a greater depth in the soil based on the proportion of postfire seedlings recruited from caches increasing with increasing fire severity (Parker 2015a). This indicates that caching behavior increases the probability of seed survival by reducing heat flow to the seed during a fire event. Second, cached seeds in this genus are almost always found as a collection of multiple fruit or seed (Moore and Vander Wall 2015; Parker 2015a); some seed may be protected by surrounding seeds in the cache that may reduce heat flow to some of the more interior or lower seed.

The objective of this research was to extend earlier work that suggests that caching improves survival of wildfire for seed of dominant plants of shrublands, in our case California chaparral and high elevation conifer–shrubland mosaics (Moore and Vander Wall 2015; Parker 2015a). Specifically, we want to quantify two potential processes that would cause cached seeds to germinate at greater rates than noncached seed after high‐intensity fires. One is clear, the importance of seed depth to escape the kill zone of a fire's heat pulse. For this goal, we will determine the upper and lower limits of killing temperatures, and what depths might be reflective of such temperatures. The second process has not been explicated in any other studies of which we have knowledge: we predict that seeds, clustered together in the form of a cache, can reduce heat flow to interior or lower seeds in the same cache. The significance of this is that within the heat pulse kill zone of the soil, more seed may survive than may be otherwise predicted. Because many fruit or seed are cached within the potential kill zone (Moore and Vander Wall 2015; Parker 2015a), and because fires are heterogeneous and this heat pulse kill zone varies among sites, this could be a significant source of seed survival. We approach these objectives experimentally using synthetically produced seed banks under controlled laboratory conditions.

## Methods and Materials

### Synthetic seed bank

To mimic heating profiles and seed bank structures found in the field, we constructed a synthetic seed bank. This consisted of a cube‐shaped metal vessel 25.5 cm square and 25.5 cm deep. To record temperatures in the soil, holes for thermocouples (K type) were drilled at 4 depths (0.5, 1.5, 2.5, and 4 cm) in two places making a total of 8 holes. In the bottom 17.5 cm of the vessel, perlite was used as an insulator to prevent heat entering the seed bank from the bottom. Perlite is a good insulator while not adding much weight to the vessel allowing for quick and easy movement in and out of the oven while the vessel is hot. The 5 cm left at the top of the vessel was filled with fine sand, which acted as the soil matrix for the synthetic soil seed bank. In this 5 cm of sand, seeds were placed at specific depths and cache sizes depending on the desired treatment. *Arctostaphylos* species occur on a wide variety of soils, from fine clays, sandstones, volcanics, granitics, sand dunes, and sandy soils as a breakdown product of sandstones or windblown dunes. We used fine sand as representative of the majority of the soils this genus is found on, and especially due to its consistency, and the absence of organic material that would potentially increase temperature variability at under high temperatures.

### Artificial fire

The challenge of mimicking fire in the laboratory is both the high temperatures and the directionality of the heat. In a wildfire, soil heating occurs strictly from top down. To achieve the desired effects, a small Thelco oven was fitted with an Omega QG Infrared Radiant Heater controlled by a Bowens [B] microlite control processer. This heating element and processor can reach temperatures of 1000°C. However, the insulation of the oven does not allow temperatures to get higher than 650°C. The heating element was fixed to the top of the oven facing down so that the heat would move downwards as it would in a natural fire, with the heating element only 5 cm from the surface of the synthetic seed bank.

**Figure 1:**
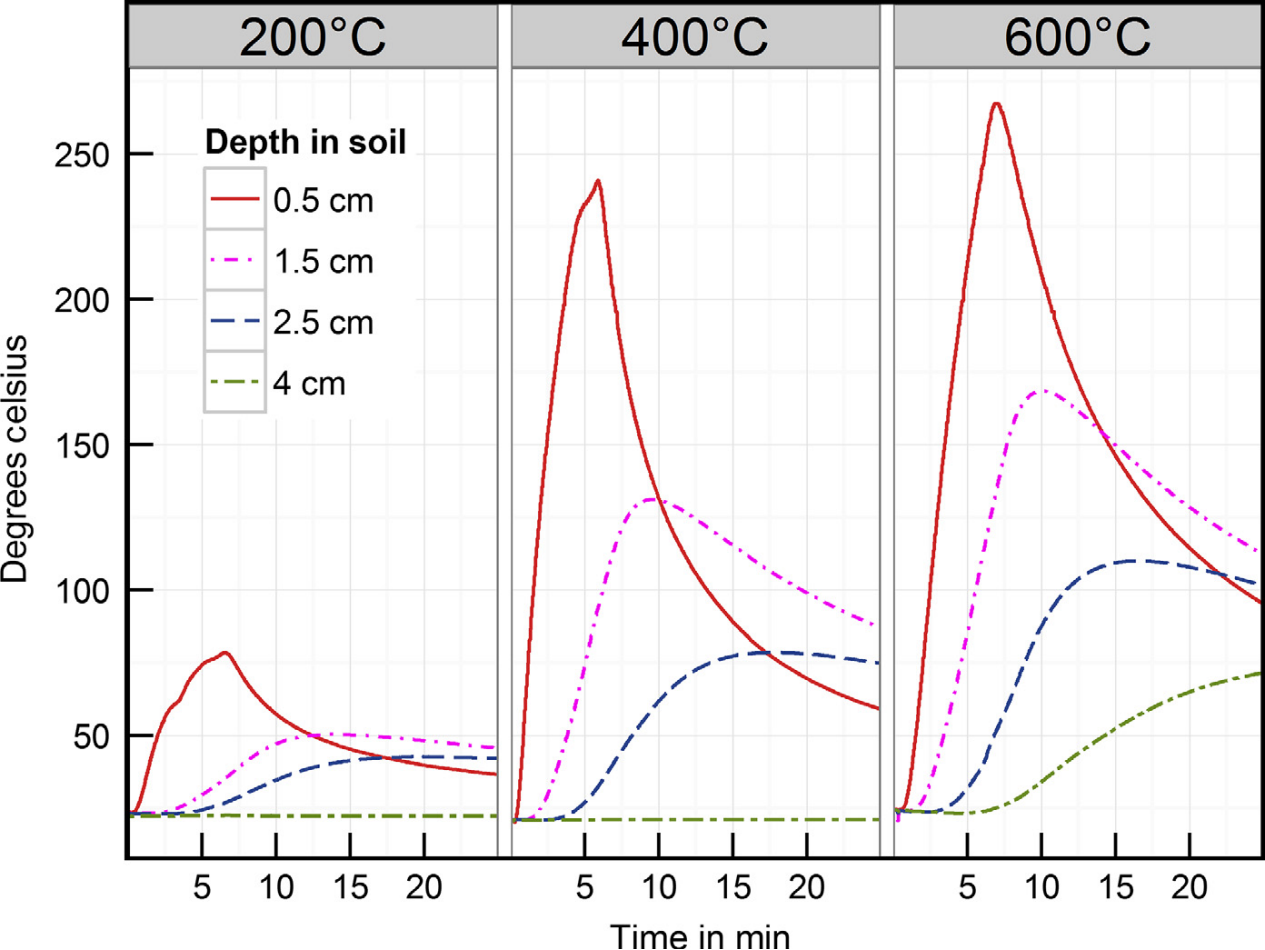
Heating profiles at different depths and surface temperatures. Heating profiles were collected from synthetic soil seed bank using thermocouples at 3 surface temperatures (200, 400, and 600°C) and 4 depths (0.5, 1.5, 2.5, and 4.0 cm).

After the synthetic seed bank was constructed, the oven was heated to the desired surface temperature before the seed bank vessel was placed in the oven. This helped ensure the seed bank was exposed to an initial flash of heat as it would be in a fire. The vessel was placed under the heating element for 5 min; it was then removed from the oven and left out at room temperature for 20 min. The heating profiles from the oven (Fig. 1) showed a close match to field observations (DeBano 1988). Thermocouples recorded temperatures during the whole 25 min. Seeds were then extracted from the synthetic seed bank by sifting the sand through a 1.68‐mm sieve.

### Viability test

After removal from the synthetic seed banks, seeds were stored in paper bags up to 2 days until analyzed. Each seed was cut in half to expose the embryo. The seeds were then soaked in a solution of TZ at the 0.01% concentration as specified in the Tetrazolium Testing Handbook (2013). After soaking for 18–24 h, they were assessed for evidence of viability under a microscope.

### Seed collection and storage

*Arctostaphylos* produces roughly globose fruit that range from 2 to 15 mm in diameter within which are up to 10 seeds (typically 6–7 seeds). Seeds as discussed in this study consist of a hard endocarp surrounding a single seed. Seeds in the fruit fit together similar to segments of an orange. Some *Arctostaphylos* species have seeds that are partially fused or fully fused together inside of the fruit, while seeds in other species are not fused together and are referred to as free seeds. Seed fusion probably increases seed survival for any given temperature, so we deliberately selected *A. viscida* for our experiments, a species that has free seeds, to insure any caching benefit would apply across the genus. The fruits of *A. viscida* were collected in the field in the South Yuba River drainage, Nevada County, California, in August 2013. Seeds with endocarps were cleaned from the rest of the fruit by lightly breaking apart the fruit in a plastic bag and winnowing off the exocarp and mealy mesocarp. Seeds were then thoroughly mixed together and were stored in a paper bag at ambient room temperature in the dark. To establish the viability of the population of collected seed, eight samples of 50 seeds were tested for viability as described above.

### Seed treatments

Each treatment investigated a specific seed depth, cache size, and surface temperature. Seeds were placed in sand in the synthetic soil seed bank at the desired depth (0.5, 1.5, 2.5, or 4.0 cm). This range of depths represents the most densely populated portion of the seed bank in the field (Keeley 1977; Parker and Kelly 1989; Odion and Davis 2000). Treatments were subjected to one of three surface temperatures: 200°C, 400°C, or 600°C. This reflects the range of surface temperatures during a chaparral fire found in the field (DeBano 1988). Seed cache sizes were 1 (no cache) or 5 or 10 seeds surrounding an experimental seed. The temperature at the depth of seed was recorded using a thermocouple during all treatments.

Seeds initially were placed at the desired level in the seed bank, and during burial, every seed was inspected for proper depth and adjusted appropriately. In the cache sizes of 5 and 10, a single treatment seed was surrounded by a “matrix” of seeds that represented the cache. Matrix seeds were marked lightly with paint to differentiate them from the treatment seed. The treatment seed was placed at the desired depth, and the matrix seeds were carefully placed around the treatment seed in a pyramid‐like structure. The seed bank vessel was then placed in the preheated oven for 5 min. After the 5 min treatment, the vessel was removed and cooled at room temperature for 20 min. Seeds were then extracted from the seed bank by sifting the soil through a 1.68‐mm sieve. Seeds were then extracted from the seed bank and processed as described above.

### Experiment 1: Model of mortality in the soil

An initial experiment was conducted on individual seeds to establish temperature limits of seed survival in the context of a seed bank after a heating event. Probability of seed survival was modeled using the independent variables: maximum temperature at seed level, surface temperature, and depth of seed. Treatments were determined by the combination of two variables: surface temperature and depth of seed. There were three surface temperatures used (200, 400, and 600°C) and four depths in the soil (0.5, 1.5, 2.5, and 4.0 cm) for a total of 12 possible treatments. In total, 30 replicates were run, 16 replicates consisting of 50 seeds per treatment and 14 consisting of 200 seeds per treatment. In total 3600 seeds were treated and analyzed for viability in the way described above.

### Experiment 2: Effect of cache size

Based on results from the mortality experiment, cache treatments were limited to 1.5 and 2.5 cm depths, depths within the heat pulse kill zone. Additionally, treatments were limited to a range of maximum temperatures experienced at the treatment seed depth between 75 and 150°C. These temperatures represent a critical range where *Arctostaphylos* seed begin to see a reduction in viability at the low end and total mortality at the high end. Temperature data collected in the previous experiments allowed us to predict what combination of surface temperatures and seed depth would provide maximum temperatures in the desired range. Seeds were placed in cache formations consisting of either 5 or 10 matrix seeds, with the treatment seed placed at the desired depth and surrounded on all four sides with the additional cache seed, and buried with the appropriate depth of sand. Each cache treatment consisted of 50 caches and was replicated 25 times (11 with caches of 5, 14 with caches of 10 seed). These were then compared to the “cache” size of 1 from the initial experiments.

### Experiment 3: How much does a seed cache reduce heat flow

In a separate experiment, thermocouples were used to measure the difference in temperatures between different cache sizes. Two thermocouples were placed at the same depth in the synthetic soil seed bank, one with a cache (seeds) surrounding the sides and top of the thermocouple, the other with no cache. This was done at all three surface temperatures at depths of 1.5 cm and 2.5 cm.

### Data analysis

All seed viability was collected as binary data (alive, dead). All seeds from a single treatment, either 50 or 200 depending, were seen as a sample and the binary data was converted into percent survival. Percent survival was then modeled using the variables of maximum temperature at seed level, depth of seed, and in the cache experiments, cache size. Data were fit using a logistic regression model, and models were compared using AIC scores. Model fit was assessed using Mcfadden's pseudo‐R‐squared and the log‐likelihood pseudo‐R‐squared. All analysis were performed in R 3.0.3 (R Core Team 2014) using the rms (Harrell 2014) package for logistic regression and the pscl (Jackman 2014) package for assessing model fit.

## Results

### Experimental seed viability

Field‐collected seed indicated low and variable seed viability. The viability for eight sets of 50 seeds ranged from 16% to 53% with an average of 32.25% (±10.26).

### Modeling survival probability using maximum temperature

**Figure 2:**
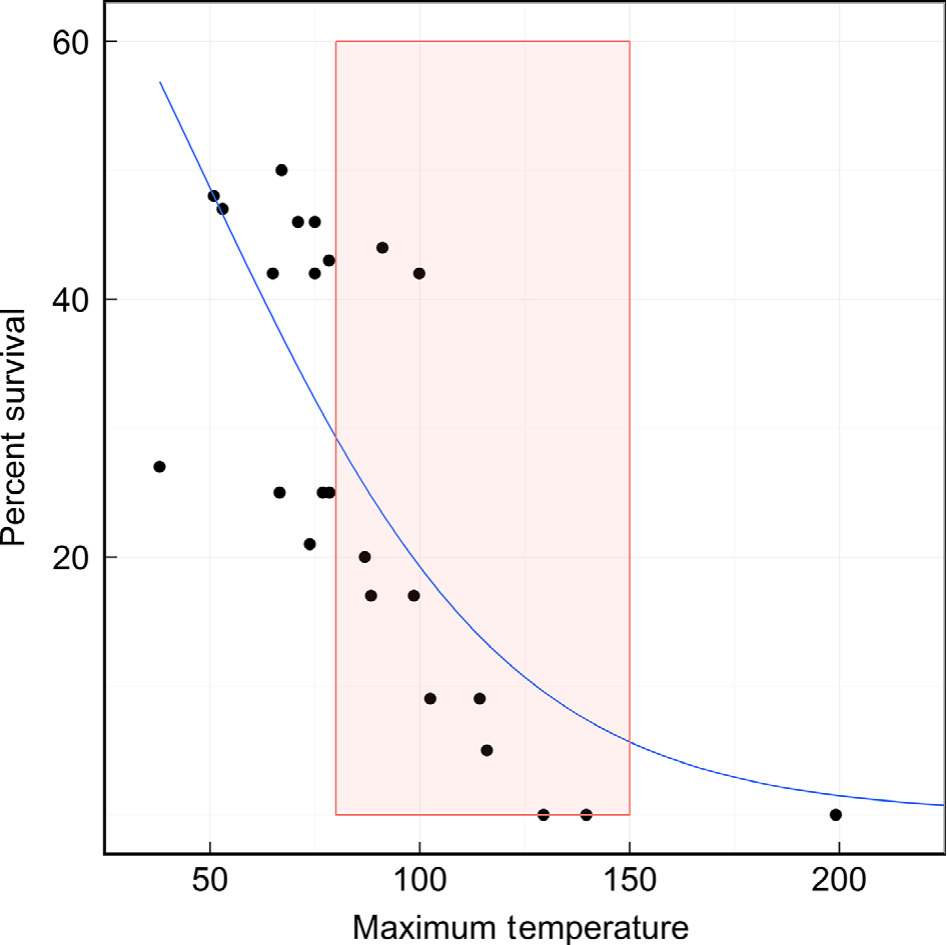
Logistic regression model modeling survival of seed using maximum temperature reached at seed depth. The red box illustrates the temperature range at which mortality begins to reduce (85°C) and where total seed mortality is reached (150°C).

The model including maximum temperature reached at seed level, depth of seed, and the interaction between maximum temperature and depth was the most informative and had the best fit to the data (Table 1). All variables in this model were significant at an alpha level of 0.05 (Table 2). Surface temperature was not used as a variable because it was not seen as a reliable measurement due to variability of the position of the feedback thermocouple controlling the heating element. The data show the probability of survival starts to reduce significantly at a maximum temperature around 85–90°C with complete mortality at 130°C. Figure 2 illustrates this relationship and shows the region of maximum temperature where cache size might have the most influence.

**Table 1:** Comparing probability of seed‐survival models. Comparing models using AIC and assessing model fit using McFadden's pseudo‐R‐squared and log‐likelihood pseudo‐R‐squared

Model	AIC	McFadden's pseudo‐R‐squared	Log‐likelihood pseudo‐R‐squared
Maximum temperature + depth + maximum temperature × depth	280.97	0.652	0.747
Maximum temperature + depth	330.76	0.587	0.672
Maximum temperature	332.56	0.582	0.666

**Table 2:** Model output for the full logistic regression model

Variable	Coefficient	Std error	*z* value	*P* value
Intercept	4.042	0.474	8.535	<2E‐16
Maximum temperature	−0.062	0.006	−11.103	<2E‐16
Depth	−1.063	0.152	−7.007	2.44E‐12
Maximum temperature × Depth	0.013	0.002	6.721	1.80E‐11

During a heating event, depth of seed was a significant factor in determining the amount of heat to which seeds were exposed. Temperatures were significantly reduced as heat moved down through the soil in the synthetic seed bank with seeds at greater depths less likely to be exposed to high temperatures that cause mortality. While this appears to be an obvious result, in the context of either a single severe but heterogeneous fire or a variable fire regime, different depths received mortality risk differentially over the range of surface temperatures. Across all surface temperatures in this experiment, heating profiles collected from the thermocouples show that temperatures at the 0.5 cm level exceeded 85°C 66% percent of the time, whereas temperatures at 4 cm never exceeded 85°C. Thus, seeds at the 0.5 cm level are at risk under most conditions whereas seeds at the depth of 4 cm and below are safe under most, if not all, conditions. In contrast, seeds at depths of 1.5 and 2.5 cm were more variable in their response to above‐ground heating conditions and thus more sensitive to changes in environmental conditions such as cache size. This experiment provided a range of temperatures 85–150°C, and seed depths 1.5 and 2.5 cm, where potential increased protection from cache size would have the most impact and be the most detectable.

### Determining the effect of cache size

**Figure 3:**
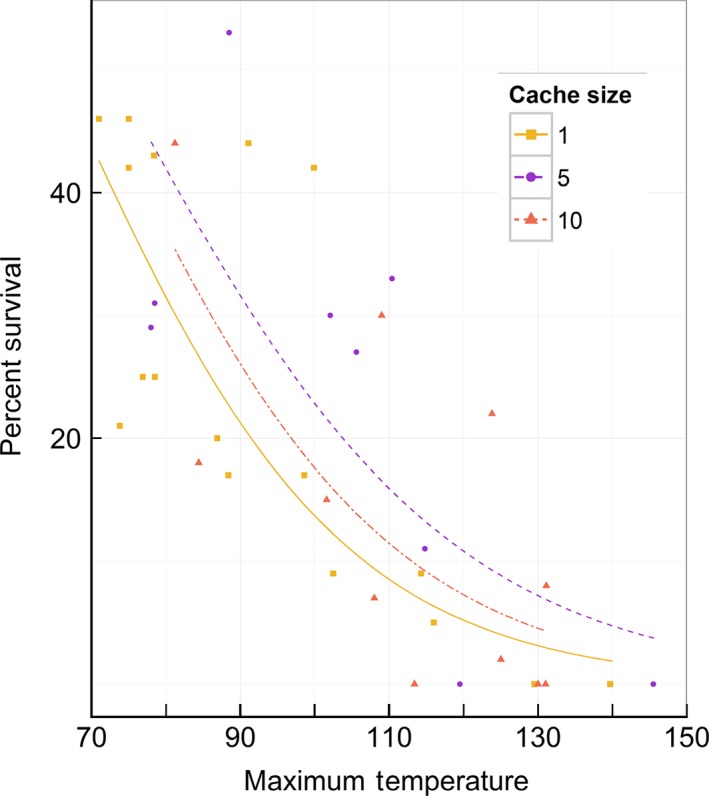
Comparing logistic regression models of seed survival grouped by cache size.

For this experiment, a majority of the data collected were in the temperature range of 75–150°C at the depth of the experimental seed and at initial of depths 1.5 and 2.5 cm. Cache size significantly improved the model (Table 3). Both cache sizes of 5 and 10 increased survival of test seed suggesting that the cluster of seeds representing the cache reduced heat flow to the treatment seed (Fig. 3).

**Table 3:** Comparing probability of seed‐survival models with and without cache as a variable. Models are compared using AIC, and model fit is assessed used McFadden's pseudo‐R‐squared and log‐likelihood pseudo‐R‐squared

Model	AIC	McFadden's pseudo‐R‐squared	Log‐likelihood pseudo‐R‐squared
Model comparison with and without cache size			
Maximum temperature + depth + cache + maximum temperature × depth	315.46	0.594	0.714
Maximum temperature + depth + cache	315.27	0.591	0.71
Maximum temperature + depth	330.42	0.566	0.678

### Quantifying the amount of heat reduction from cache

**Figure 4:**
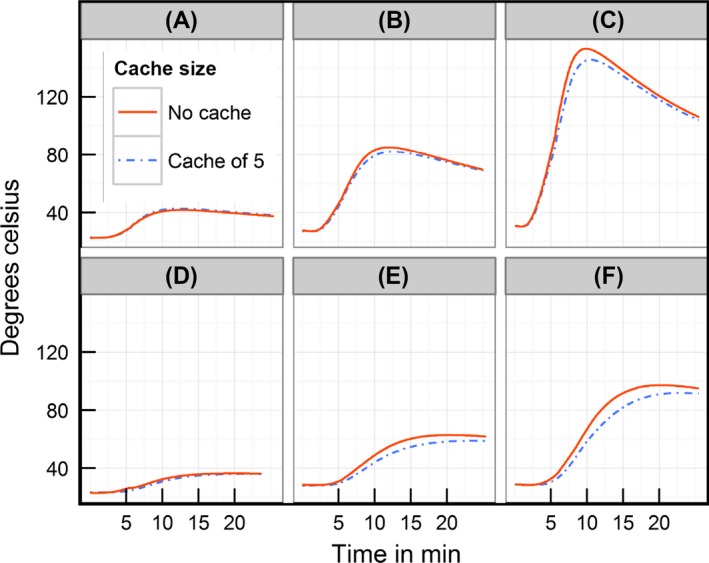
Comparing heating profile of cache of 5 and no cache across 3 surface temperatures (200, 400, and 600°C) and 2 depths (1.5 and 2.5 cm). (A) 200°C at 1.5 cm; (B) 400°C at 1.5 cm; (C) 600°C at 1.5 cm; (D) 200°C at 2.5 cm; (E) 400°C at 2.5 cm; (F) 600°C at 2.5 cm.

**Figure 5:**
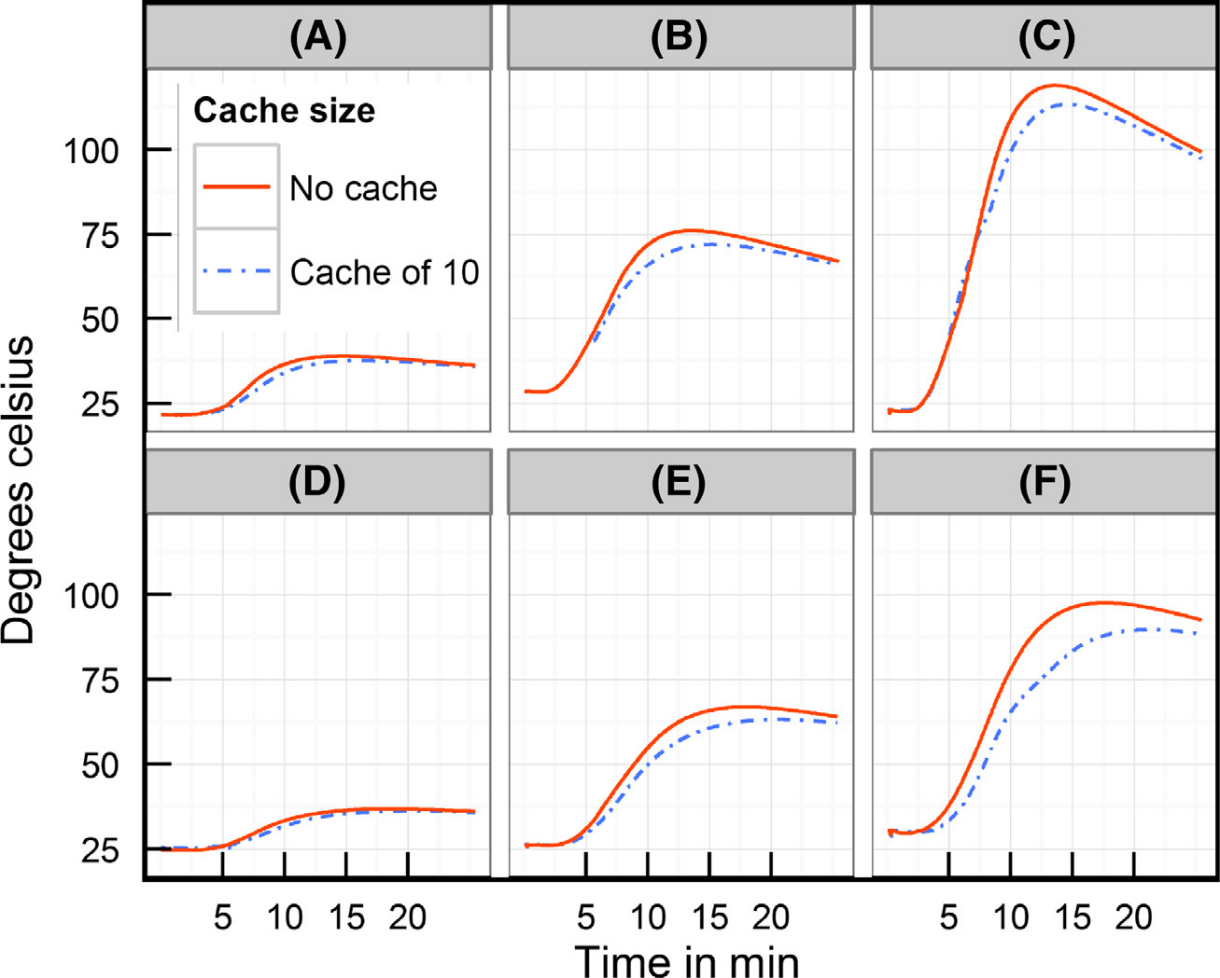
Comparing heating profile of cache of 10 and no cache across 3 surface temperatures (200, 400, and 600°C) and 2 depths (1.5 and 2.5 cm). (A) 200°C at 1.5 cm; (B) 400°C at 1.5 cm; (C) 600°C at 1.5 cm; (D) 200°C at 2.5 cm; (E) 400°C at 2.5 cm; (F) 600°C at 2.5 cm.

Thermocouples surrounded by seed to mimic a cache recorded lower temperatures than thermocouples without seed. The difference in temperature was dependent on the depth of the thermocouples and the size of the cache. Figures 4 and 5 illustrate the temperature difference between cache and no cache with cache size of 5 and 10, respectively. The biggest reduction in maximum temperatures reached was at the higher temperatures produced by a surface temperature of 600°C. For the cache size of 5, there was a reduction of 7.6°C at 1.5 cm and 5.4°C at 2.5 cm. For the cache of 10, there was a reduction of 5.7°C at 1.5 cm and 8°C at 2.5 cm at a surface temperature of 600°C.

## Discussion

Rodent‐caching behavior increases the probability of *Arctostaphylos* seed survival in the seed bank during a heating event by two different processes, by depth and by clusters of seed in caches disrupting heat flow. In our experiments, seeds at greater depths in the soil and seeds within caches have an increased chance of survival. Differences in caching behavior within and among rodents provide variation in seed depth and configuration (Moore and Vander Wall 2015; Parker 2015a). Consequently, this structuring of the seed bank potentially expands seed survival under a range of fire conditions and fire regimes, for what might otherwise be a bottleneck in *Arctostaphylos* life history. Thus, more generally, in any vegetation prone to a wildfire regime, rodent caching could be a significant dispersal or survival mutualism. These results also indicate mutualisms may depend on disturbance regimes (Parker 2015a), in contrast to the disruption or breakdown of mutualisms by anthropogenic disturbances (e.g., Kiers et al. 2015).

*Arctostaphylos* populations persist under chronic pressure from fire due to persistent soil seed banks that lay dormant until stimulated to germinate by fire. Although seeds require chemicals from smoke generated by fire to trigger germination, seeds also experience an increase in mortality at temperatures above 85°C. Seed survival of fire is largely regulated by the insulating properties of the soil and with depth in the soil; seeds are protected from high temperatures. While the specifics would vary among soil types and fire severity, in this study the probability of seed survival during a heating event rapidly increases the deeper the seed is in the soil seed bank. During heating treatments, for example, across all surface treatment temperatures, the soil depth of 0.5 cm reached or exceeded 85°C 66% of the time whereas temperatures at a depth of 4 cm never exceeded 85°C, illustrating the relative risk seeds face at different depths. Rodent caching provides a mechanism for seed to reach lower levels in the soil, thus increasing the survival of seed survival and probability of population persistence over time. There is a limit, however, to how deep seeds can be in the soil before germinating seeds fail to reach the surface with the amount of energy stored in the seed (e.g., Bond et al. 1999). Thus, with depth, seed survival and germination have a positive gradient with respect to heat but a negative gradient in their ability to reach the surface. One additional major risk *Arctostaphylos* species face is a decreased fire‐free interval, too short to get enough seeds deep enough in the soil to survive the next fire, a phenomenon called immaturity risk (Zedler 1995; Keeley et al. 2005). Because *Arctostaphylos* seed is generally too large to abiotically migrate into the soil (Parker 2015a,b), in the context of immaturity risk in young stands, seed‐caching rodents may be crucial for *Arctostaphylos* ecology.

The behavior of caching clusters of seed also increases probability of seed survival. Seeds surrounded by other seeds in a cache had an increase in survival during a heating event with killing temperatures by roughly 10% over seeds not in a cache; but given the overall low viability of the seed pool, effectively survival increased almost 25%. Cached seeds allow for survival at depths closer to the soil surface, thus ameliorating some of the impacts of fire severity. Interestingly, the data show that the cache size of 5 had greater survival than the cache of 10; this is probably due to low viability of the underlying seed population and a sample size too small to detect the difference between caches (5 and 10) versus no cache (1) or indicates only small differences among the cache sizes (5 and 10, in this case). The low viability of seeds may by itself increase handling time for rodents when retrieving seed, increasing the chances some seed will remain in caches. Increasing the density of seedling establishment is critical for these species given the difficulty of surviving both summer drought and herbivory (Parker et al. 2015).

Cache sizes in this study also only reflect the lower end of cache sizes found in the field. Parker (2015a) found that when *Arctostaphylos* fruits were offered in the field, rodent cache sizes were on average made up of 4.1 fruits (ranging from 1 to 12), each fruit containing an average of seven seeds, equaling an average of 28–30 seeds per cache. Moore and Vander Wall (2015) offered *Arctostaphylos* seed separated from the rest of the fruit and found caches ranging from 1 to 76 seeds, with a median of 8.5 seed. This would suggest that the effect of caching on increased seed survival may be stronger in field conditions. In addition, *Arctostaphylos viscida*, the species used in these experiments, has seed that is smaller than most other species in the genus. Seed size affects survival ability during a fire event presumably because thicker endocarps in larger seed sizes provides greater insulation to the embryo and thus improves the chance of survival (Keeley 1977). Response to caching behavior may differ among *Arctostaphylos* species due to variation in seed size.

*Arctostaphylos* is not limited by the amount of seed it produces (Keeley 1987; Parker 2015a,b), but by the bottleneck of seed surviving a fire event. If rodents leave sufficient seed in the seed bank while structuring the seed bank to favor survival of seed during a fire, then these groups of species mutually benefit each other. The conditional mutualism between *Arctostaphylos* and caching rodents also is contingent on biotic and abiotic conditions (Bronstein 1994; Billick and Tonkel 2003; Klinger and Rejmánek 2010). Many studies report rodent predation as a major limitation to *Arctostaphylos* recruitment (Jameson 1952; Keeley and Hays 1976; Parker and Kelly 1990). However, *Arctostaphylos* seed bank numbers remain relatively consistent among years despite an annual pulse of seed production (Keeley 1987), suggesting rodent seed predation has a lower limit and that scatter hoarders replenish seed lost to consumption. Differential responses among rodent species to abiotic factors, such as climate and fire regimes, or biotic factors, such as predators, possibly contribute to an averaging effect. Relatively large seed bank densities (Keeley 1977; Parker and Kelly 1989; Parker 2015b) and postfire seedling emergence from caches (Parker 2015a) indicates a positive net outcome for plants interacting with scatter‐hoarding rodents.

In summary, our findings suggest a multifaceted mutualism in the relationship of scatter‐hoarding rodents and *Arctostaphylos* species. The heterogeneity of any fire and variation in soil texture result in variable displacements of lower depth boundaries of the heat pulse kill zone within a particular stand and across landscapes. Thus, in some circumstances, the shallower caches of smaller rodents will be quite effective for seed survival, while with greater fire severities, only the deeper caches may survive. Within the heat pulse kill zone, seeds within caches may differentially survive as well. As recently discussed by Bronstein (2015), scatter‐hoarding rodents and *Arctostaphylos* seed reveal the complexities of mutualism categories. Rodent caching acts not only as directed dispersal to safe sites, in this case vertically down into the soil, but additionally the clustering of seed in caches also acts as a protection mutualism because it further protects some seed from killing temperatures by disrupting the heat pulse. Additionally, this study indicates the scatter‐hoarding plant mutualism depends on the context of disturbance regimes. Evolving understandings of the multiple dimensions of such relationships allow researchers and land managers to ask more accurate questions and make ecologically sound decisions in the future.

## Conflict of Interest

None declared.
